# Letter to the Editor regarding “Ferroptosis is involved in the IL-9-induced intestinal barrier injury in sepsis: an experimental animal and translational study”

**DOI:** 10.1097/JS9.0000000000003584

**Published:** 2025-10-10

**Authors:** Peiqi Ying, Yingsong Xu, Yan Li

**Affiliations:** a Department of Intensive Care Unit, Beilun People’s Hospital, Ningbo, Zhejiang Province, China; b The First Affiliated Hospital of Dalian Medical University, Dalian, China

*Dear Editor,*

We read with great interest the recent article by Sun *et al*^[1]^ titled “Ferroptosis is involved in the IL-9-induced intestinal barrier injury in sepsis: an experimental animal and translational study.” This study used clinical patient serum samples and animal models of sepsis to analyze the relationship between IL-9, ferroptosis, and intestinal injury in sepsis. We greatly appreciate and recognize the study’s conclusion that increased expression of IL-9 promotes ferroptosis to further induce sepsis. The findings of this study are novel and have greater research implications for the clinical diagnosis of intestinal injury in sepsis. However, our meticulous study of the article identified some problems that need to be discussed with the authors. This article has been published in accordance with The TITAN Guideline conducted a transparency review of AI reporting^[2]^.

Firstly, the study included 56 patients suffering from sepsis and 18 non-sepsis patients from the ICU ward as a control group. We found that for each of these 74 patients, each patient had a serious underlying disease. In particular, the 18 patients in the control group suffered from six different underlying diseases. Whether this difference in underlying diseases could affect IL-9 expression^[3]^. We encourage the authors to address in their article whether there is a potential relationship between the patients’ underlying diseases and IL-9.

Secondly, the animal experiments in this study, by constructing a sepsis model and imposing different treatments, analyzed the effect of changes in IL-9 on ferroptosis in sepsis. The grouping of animal models was reasonable and the results were more obvious. However, we found that changes in IL-9 expression were not detected in the animal experiments to demonstrate the success of the imposed interventions. We encourage the authors to add this part of the data. In addition the study used AAV viral particles to regulate gene expression in the animal model and ferroprotease inhibitors were administered to inhibit iron death. We suggest that the authors state in the Methods section whether the control and untreated sepsis groups, were given empty AAV virus particles and drug solvents to exclude the effect of the corresponding confounding factors.

Finally, the study analyzed potential changes in the intestinal epithelial tissue of animal models under different interventions by electron microscopy and multiple staining techniques. We found that Figures 7 and 8 have corresponding scale bars for each figure, which is necessary. However, we encourage the authors to increase the magnification of the images in Figure Legend to increase the readability of the article.

In summary, this study obtained that increased IL-9 may aggravate intestinal injury in sepsis by affecting the occurrence of ferroptosis and thus exacerbating sepsis through clinical patient and animal model data. We appreciate the research methodology of the study and recognize the conclusions obtained^[4,5]^. However, we found that there are still some minor problems in the article through detailed research, and we hope that the authors can make improvements.

## Data Availability

Not applicable.
